# Analysis of Anxiety or Depression and Long-term Mortality Among Survivors of Out-of-Hospital Cardiac Arrest

**DOI:** 10.1001/jamanetworkopen.2023.7809

**Published:** 2023-04-12

**Authors:** Juncheol Lee, Yongil Cho, Jaehoon Oh, Hyunggoo Kang, Tae Ho Lim, Byuk Sung Ko, Kyung Hun Yoo, Sang Hwan Lee

**Affiliations:** 1 Department of Emergency Medicine, College of Medicine, Hanyang University, Seoul, Republic of Korea

## Abstract

**Importance:**

The recent American Heart Association guidelines added a sixth link in the chain of survival highlighting recovery and emphasized the importance of psychiatric outcome and recovery for survivors of out-of-hospital cardiac arrest (OHCA). The prevalence of psychiatric disorders among this population was higher than that in the general population.

**Objective:**

To examine the prevalence of depression or anxiety and the association of these conditions with long-term mortality among individuals who survive OHCA.

**Design, Setting, and Participants:**

A longitudinal population-based cohort study was conducted to analyze long-term prognosis in patients hospitalized for OHCA between January 1, 2005, and December 31, 2015, who survived for 1 year or longer. Patients with cardiac arrest due to traumatic or nonmedical causes, such as injuries, poisoning, asphyxiation, burns, or anaphylaxis, were excluded. Data were extracted on depression or anxiety diagnoses in this population within 1 year from the database of the Korean National Health Insurance Service and analyzed April 7, 2022, and reanalyzed January 19 to 20, 2023.

**Main Outcomes and Measures:**

Follow-up data were obtained for up to 14 years, and the primary outcome was long-term cumulative mortality. Long-term mortality among patients with and without a diagnosis of depression or anxiety were evaluated.

**Results:**

The analysis included 2373 patients; 1860 (78.4%) were male, and the median age was 53.0 (IQR, 44.0-62.0) years . A total of 397 (16.7%) patients were diagnosed with depression or anxiety, 251 (10.6%) were diagnosed with depression, and 227 (9.6%) were diagnosed with anxiety. The incidence of long-term mortality was significantly higher in the group diagnosed with depression or anxiety than in the group without depression or anxiety (141 of 397 [35.5%] vs 534 of 1976 [27.0%]; *P* = .001). With multivariate Cox proportional hazards regression analysis, the adjusted hazard ratio of long-term mortality for total patients with depression or anxiety was 1.41 (95% CI, 1.17-1.70); depression, 1.44 (95% CI, 1.16-1.79); and anxiety, 1.20 (95% CI, 0.94-1.53).

**Conclusions and Relevance:**

In this study, among the patients who experienced OHCA, those diagnosed with depression or anxiety had higher long-term mortality rates than those without depression or anxiety. These findings suggest that psychological and neurologic rehabilitation intervention for survivors of OHCA may be needed to improve long-term survival.

## Key Points

**Question:**

Is depression or anxiety associated with increased long-term mortality among patients after out-of-hospital cardiac arrest?

**Findings:**

In this cohort study using claims from the Korean National Health Insurance Service database, 2373 patients with out-of-hospital cardiac arrest were followed up for up to 14 years. Patients diagnosed with depression or anxiety had an approximately 40% higher long-term mortality rate than those without such psychiatric disorders.

**Meaning:**

The findings of this study suggest that psychological and neurologic rehabilitation intervention for survivors of out-of-hospital cardiac arrest may be needed to improve long-term survival.

## Teaser

This cohort study examines the prevalence of depression or anxiety and the association of these conditions with mortality in long-term follow-up of individuals with out-of-hospital cardiac arrest.

## Introduction

The incidence rate of out-of-hospital cardiac arrest (OHCA) is 84.0 per 100 000 population; OHCA is a major public health problem and a leading cause of mortality and morbidity.^1,2^ The rate of survival with good neurologic outcomes after OHCA has increased in recent decades.^2,3^ As a result of the increase in the rate of good prognosis of patients with OHCA, the long-term outcomes would also be increased. Surviving patients could develop neurologic sequelae caused by both initial anoxia and subsequent ischemia-reperfusion injury, and such sequelae could affect their physical, cognitive, and psychosocial characteristics.^4,5,6^

Many previous studies assessed survival rates or neurologic prognoses of patients with OHCA using tools such as the Cerebral Performance Category scale.^7,8,9^ However, according to the European Resuscitation Council and European Society of Intensive Care Medicine guidelines, it is also important to perform functional assessments of nonphysical impairments and screen for cognitive and emotional problems in the long term in patients with OHCA.^10^ In 2020, a sixth link in the chain of survival highlighting recovery was added to the American Heart Association guideline to emphasize the importance of recovery and survivorship in resuscitation outcomes.^5^ Consequently, studies have reported the prevalence of depression and anxiety among patients after OHCA and changes in health-related quality of life due to psychiatric disorders.^11,12,13,14,15^ A systematic review and meta-analysis recently reported that the prevalence of psychiatric disorders in survivors of OHCA was higher than that in the general population, stressing the importance of improving physical and mental outcomes in individuals who experience OHCA.^16^

However, to our knowledge, long-term mortality among surviving individuals with psychiatric disorders after OHCA has not yet been reported. In the present study, we aimed to investigate the association between long-term mortality and psychiatric disorders, such as depression and anxiety, among patients after OHCA.

## Methods

### Data Sources and Setting

We conducted a population-based cohort study and extracted data from the database of the Korean National Health Insurance Service (NHIS), which is a nationwide single-payer health program in South Korea. South Korea provides medical insurance coverage to almost all citizens, covering approximately 50 million people according to the NHIS.^17^ Each hospital visit is reported to the claims database, which includes diagnostic codes based on the World Health Organization’s *International Statistical Classification of Diseases and Related Health Problems, 10th Revision* (*ICD-10*). The NHIS database contains diagnosis codes for clinic outpatients as well as patients admitted to hospitals. The NHIS database also includes inpatient and outpatient medical histories, patient demographic characteristic data, diagnoses, procedures, drug prescriptions, and dates of death.^18^ Cause of death data were obtained from Statistics Korea, which is merged with the NHIS database. All the data were analyzed after deidentification, and cause of death was classified based on *ICD-10* codes. In South Korea, fees for treatment at an emergency department (ED) are charged to all patients who visit the ED for emergency situations, such as cardiac arrest. The NHIS covers the ED management fee in part for patients with medical insurance and in full for those with medical aid. This study was approved by the institutional review board of Hanyang University Hospital, and a waiver for informed consent was granted because data were deidentified. This study followed the Strengthening the Reporting of Observational Studies in Epidemiology (STROBE) reporting guideline.

### Study Design and Population

We conducted a longitudinal cohort study to analyze long-term prognosis. We extracted the data of patients with a primary diagnosis of cardiac arrest (*ICD-10* code I46.x) for the first time from January 1, 2005, to December 31, 2015. We excluded patients with in-hospital cardiac arrest without a code for ED management fees for cardiac arrest or a primary diagnosis code for cardiac arrest. To confirm the definition of OHCA, we reviewed the medical records of 252 patients who visited a tertiary hospital and had a code for ED management fees and a primary diagnosis code for cardiac arrest (*ICD-10* code I46.x). The positive predictive value of this definition was 92.1%.^19^ The exclusion criteria were as follows: (1) age younger than 18 years, (2) survival for less than 1 year, or (3) cardiac arrest due to traumatic or nonmedical causes (*ICD-10* S, T codes), such as injuries, poisoning, asphyxiation, burns, or anaphylaxis. In addition, we excluded patients who had a code for depression or anxiety within 3 years of cardiac arrest as a washout period to target newly diagnosed patients after OHCA.

### Variables

Follow-up data were obtained for up to 14 years (until December 31, 2018) and were analyzed, and the primary outcome was long-term cumulative mortality. In addition, the data of patients diagnosed with depressive or anxiety disorder within 1 year were extracted, and the diagnoses of depression and anxiety were confirmed in patients who visited an outpatient clinic or hospital on at least 1 occasion by the presence of *ICD-10* diagnostic codes F32.x (depression) and/or F41.x (anxiety). The date on which the diagnostic code was first entered was regarded as the time of diagnosis. Then, we compared the long-term survival rates among the total group diagnosed with depression or anxiety, group diagnosed with depression, group diagnosed with anxiety, and the undiagnosed group.

The independent variables included age category, sex, and the Charlson Comorbidity Index (CCI) score. The CCI score was calculated at the time of diagnosis within 1 year before the index date using the Quan algorithm.^20,21^

Several variables were included in the analysis. First, in this study, we examined long-term mortality in patients with newly diagnosed depression or anxiety among those who survived after OHCA. However, additional analysis was conducted on whether a diagnosis of depression or anxiety before OHCA was associated with long-term mortality. Second, there may be many factors associated with long-term mortality in patients who survived after OHCA. Among them, we additionally classified the diagnosis codes of myocardial infarction, and the prescription codes of antipsychotics, antidepressants, and sedatives and analyzed the long-term mortality of patients including these variables. Third, we analyzed the causes of mortality of patients who survived after OHCA. Accordingly, we further classified them into cardiovascular mortality, noncardiovascular mortality, and injury.

### Statistical Analysis

The data were analyzed using R, version 4.0.4 (R Foundation for Statistical Computing) and SAS, version 9.4 (SAS Institute Inc). Analysis was conducted on April 7, 2022, and again January 19-20, 2023. Anderson-Darling tests were performed for variables with normal distributions in all data sets. Descriptive statistics were used to describe the baseline characteristics of the patients. Categorical variables are presented as frequencies and percentages and continuous variables as medians (IQRs) or means (SDs) . Independent *t* tests or Mann-Whitney tests were used for comparisons of continuous variables; the Fisher exact test was used for categorical variables. The cumulative mortality was estimated by the Kaplan-Meier method.

Multivariable Cox proportional hazards regression analyses were used to identify predictors of long-term mortality. Multivariable regression analysis was performed separately for each group, and the results are presented as adjusted hazard ratios (aHRs) and 95% CIs. With 2-sided, unpaired testing, differences were considered statistically significant at *P* < .05.

## Results

### Baseline Characteristics

**Figure 1.**
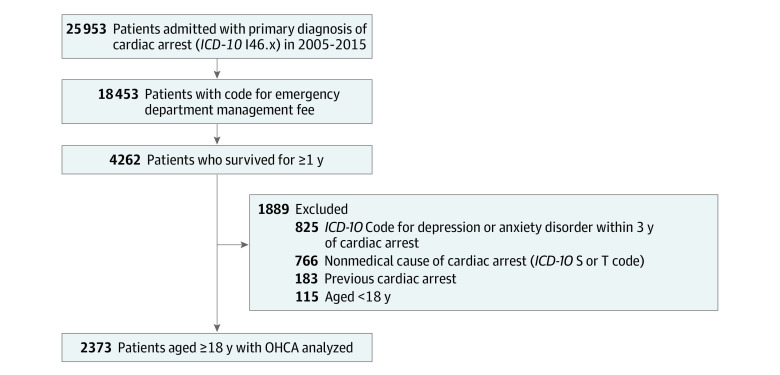
Included Patients With Out-of-Hospital Cardiac Arrest (OHCA) *ICD-10* indicates *International Statistical Classification of Diseases and Related Health Problems, 10th Revision*.

We enrolled 2373 patients after OHCA who had survived for 1 year or longer (Figure 1); median age was 53.0 (IQR, 44.0-62.0) years, 513 (21.6%) were women, and 1860 (78.4%) were men. The median follow-up was 5.1 years (IQR, 3.6-7.2 years). The baseline characteristics of the groups diagnosed and not diagnosed with depressive or anxiety disorder are summarized in Table 1. A total of 1976 patients did not receive a diagnosis of depression or anxiety, 397 (16.7%) patients were diagnosed with depression or anxiety, 251 (10.6%) were diagnosed with depression, and 227 (9.6%) were diagnosed with anxiety. The baseline characteristics of the groups diagnosed and not diagnosed with depressive disorder and diagnosed and not diagnosed with anxiety disorder are summarized in eTable 1 and eTable 2 in Supplement 1. There were no significant differences in age category, sex, or CCI score between the groups. The incidence of long-term mortality was higher in the group diagnosed with depression or anxiety than in the group not diagnosed with depression or anxiety (141 of 397 [35.5%] vs 534 of 1976 [27.0%]; *P* = .001).

**Table 1.** Baseline Characteristics of the Study Population With Depression or Anxiety Disorder

Characteristic	Patients with OHCA, No. (%)			*P* value^a^
	Total (N = 2373)	Without depression or anxiety (n = 1976)	With depression or anxiety (n = 397)	
Age, y				
18-39	426 (18.0)	343 (17.4)	83 (20.9)	.27
40-49	519 (21.9)	430 (21.8)	89 (22.4)	
50-59	694 (29.2)	575 (29.1)	119 (30.0)	
60-69	432 (18.2)	374 (18.9)	58 (14.6)	
70-79	222 (9.4)	185 (9.4)	37 (9.3)	
≥80	80 (3.4)	69 (3.5)	11 (2.8)	
Sex				
Male	1860 (78.4)	1560 (78.9)	300 (75.6)	.14
Female	513 (21.6)	416 (21.1)	97 (24.4)	
CCI score				
0	697 (29.4)	588 (29.8)	109 (27.5)	.91
1	710 (29.9)	590 (29.9)	120 (30.2)	
2	498 (21.0)	412 (20.9)	86 (21.7)	
3	270 (11.4)	223 (11.3)	47 (11.8)	
≥4	198 (8.3)	163 (8.2)	35 (8.8)	
Long-term death	675 (28.4)	534 (27.0)	141 (35.5)	.001

Abbreviations: CCI, Charlson Comorbidity Index; OHCA, out-of-hospital cardiac arrest.

^a^ Findings significant at *P* < .05. The 2 groups were compared using the Fisher exact test for categorical variables.

### Long-term Mortality According to the Diagnosis of Depression or Anxiety

**Figure 2.**
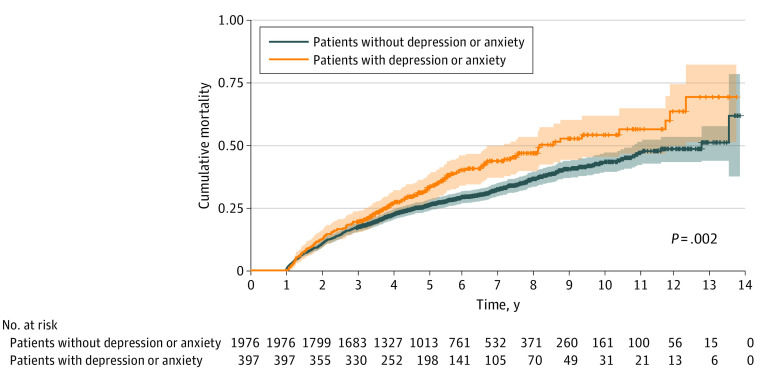
Long-term Mortality Among Patients With Out-of-Hospital Cardiac Arrest (OHCA) With and Without Depression or Anxiety Since patients who survived more than 1 year after OHCA were targeted, the first year was excluded from this analysis.

The cumulative mortality rate of patients diagnosed and not diagnosed with depressive or anxiety disorder is presented in Figure 2; the cumulative mortality was significantly higher in the individuals diagnosed with depressive or anxiety disorder (long term death, 141 of 397 [35.5%] vs. 534 of 1976 [27.0%]; *P* = .002). The cumulative mortality rates among patients diagnosed and not diagnosed with depression and among patients diagnosed and not diagnosed with anxiety are presented in the eFigure in Supplement 1. The cumulative mortality rate was significantly higher in the group diagnosed with depression (long term death, 94 of 251 [37.5%] vs. 581 of 2122 [27.4%]; *P* = .008) than in the group without a diagnosis of depression, but there was no significant difference between the group diagnosed with anxiety and the group without a diagnosis of anxiety (long term death, 72 of 227 [31.7%] vs. 603 of 2146 [28.1%]; *P* = .18).

We constructed a multivariate Cox proportional hazards regression model to assess the long-term mortality among patients with OHCA (Table 2). When other known associations were adjusted for age, sex, and CCI score, the aHR of long-term mortality among the total number of patients with depression or anxiety was 1.41 (95% CI, 1.17-1.70); patients with depression, 1.44 (95% CI, 1.16-1.79); and patients with anxiety, 1.20 (95% CI, 0.94-1.53).

**Table 2.** Multivariable Analysis to Identify Association Between Psychiatric Disorders and Long-term Mortality After OHCA

Disorder	HR (95% CI)	
	Crude	Adjusted^a^
Depression or anxiety	1.35 (1.12-1.62)	1.41 (1.17-1.70)
Depression	1.34 (1.08-1.67)	1.44 (1.16-1.79)
Anxiety	1.18 (0.93-1.51)	1.20 (0.94-1.53)

Abbreviations: HR, hazard ratio; OHCA, out-of-hospital cardiac arrest.

^a^ Adjusted variables were age category, sex, and Charlson Comorbidity Index score.

In addition, we analyzed the outcomes of the excluded patients who had an *ICD-10* code for depression or anxiety within 3 years before OHCA. We performed a multivariable analysis that included this group. As a result, there was no significant difference in the long-term mortality rate of patients who had already been diagnosed with depression or anxiety within 3 years before OHCA compared with those without depression or anxiety (aHR, 1.06; 95% CI, 0.91-1.23). However, the long-term mortality rate among patients diagnosed with depression or anxiety after OHCA was significantly higher (aHR 1.41; 95% CI, 1.17-1.70). Patients with only depression and those with only anxiety disorder within 3 years before OHCA were analyzed separately. The results also showed no significant difference in long-term mortality compared with patients without psychiatric disorders (depression: aHR, 1.09; 95% CI, 0.92-1.29 vs anxiety: aHR, 0.97; 95% CI, 0.83-1.13).

We also classified the causes of death for individuals who survived OHCA as cardiovascular mortality, noncardiovascular mortality, and injury. A total of 272 patients (14.4%) died of cardiovascular-associated causes after OHCA. Fifty-seven of 397 patients (14.2%) with depression or anxiety and 215 of 1976 individuals (10.9%) without depression or anxiety died. In a multivariable analysis including the group of patients with cardiovascular mortality, the aHR of patients with depression or anxiety was 1.41 (95% CI, 1.05-1.89). Noncardiovascular mortality accounted for the deaths of 84 of 397 (21.2%) patients with and 319 of 1976 (16.1%) patients without depression or anxiety. In multivariable analysis, the aHR of patients with depression or anxiety was 1.41 (95% CI, 1.11-1.80). A total of 24 patients died due to injury, 7 of 397 (1.8%) with and 17 of 1976 (0.9%) without depression or anxiety. In multivariable analysis, the aHR of patients with depression or anxiety was 2.34 (95% CI, 0.97-5.68).

Because new problems, such as myocardial infarction, or new medications, such as antidepressants, may have contributed to a late onset of mortality, we additionally classified the diagnosis codes of myocardial infarction and the prescription codes of antipsychotics, antidepressants, and sedatives. We performed a multivariable analysis including the groups of patients with those diagnosis codes or prescription codes. The aHR of patients with depression or anxiety was 1.63 (95% CI, 1.35-1.97), the aHR of patients with depression was 1.57 (95% CI, 1.26-1.95), and the aHR of those with anxiety disorder was 1.47 (95% CI, 1.14-1.88).

## Discussion

To our knowledge, this was the first population-based study to investigate the association between long-term mortality and psychiatric disorders among patients after OHCA. The patients diagnosed with depression or anxiety had a 1.41 times higher long-term mortality rate than those without such psychiatric disorders. The patients diagnosed with depression had a significantly higher long-term mortality rate than those without the diagnosis (aHR, 1.44), whereas there was no significant difference in the long-term mortality rate between patients diagnosed with anxiety and those without this diagnosis.

Of the 2373 patients enrolled in this study, 397 (16.7%) were diagnosed with anxiety or depressive disorder within 1 year after OHCA. Previous studies have reported that the prevalence of psychiatric disorders, such as depression and anxiety, is high among patients surviving cardiac arrest, and Yaow et al^16^ performed a systematic review and meta-analysis of such studies. In the general population, the prevalence rates were 12.9% for depressive disorder, 7.3% for anxiety disorder, and 3.9% for posttraumatic stress disorder; among patients who survived cardiac arrest, the prevalence rates were 19.0% for depressive disorder, 26.0% for anxiety disorder, and 20% for posttraumatic stress disorder. In addition, they found that the prevalence of depression and anxiety in patients who survived OHCA increased over time. Specifically, the prevalence of depression increased from 17.0% at 6 months to 30.0% at 12 months and the prevalence of anxiety increased from 34.0% at 6 months to 38.0% at 12 months. The difference in prevalence between this study and previous studies might be explained by the fact that the present study included only patients diagnosed in hospitals. Postcardiac arrest syndrome is estimated to affect both the physiologic and psychological aspects of long-term prognosis in survivors. The unpredictability and severity of OHCA can cause severe life disruptions for patients.^22,23^ The neurologic deficit caused by ischemia-reperfusion injury following OHCA can create challenges in accomplishing previously simple daily tasks without assistance.^23,24^ These realities and arduous transformations can cause extreme limitations and despondency. Such life changes can result in a decrease in health-related quality of life, which affects psychological distress.^11^ Most patients with OHCA receive immediate therapy in the intensive care unit after the return of spontaneous circulation. After receiving intensive care unit treatment, new or worsening physical and cognitive or mental disorders are referred to as postintensive care syndrome, which is estimated to affect up to one-third of survivors after intensive care unit stays.^25^ Hatch et al^26^ reported that, among patients who received intensive care unit treatment following critical illness, those with depression had an approximately 50% higher 2-year mortality rate than those without depression.

The increase in the prevalence of depression and anxiety in patients surviving OHCA is important because the increase may be a factor in a higher prevalence of psychiatric disorders and is associated with outcomes such as mortality. Cuijpers and Smit^27^ reported that the overall relative risk of mortality in patients with depression was 1.81 (95% CI, 1.58-2.07) compared with those without depression, and depression should be considered a life-threatening disorder. In addition, Zivin et al^28^ reported that the HR of patients with depression for 3-year mortality was 1.17 (95% CI, 1.15-1.18), and depression was associated with an increased risk of death due to nearly all major medical causes, regardless of the presence or absence of multiple primary risk factors. Cardiac disease is one of the factors contributing to the high mortality rate among patients with depression. It was reported that patients experiencing psychological distress had a higher incidence of myocardial infarction and worse survival and prognosis outcomes following myocardial infarction than their counterparts.^29,30^

Previous studies have reported an increase in the incidence of psychological disorders in individuals following OHCA^11,15,31^ but have not noted an association between psychological disorders and mortality in this population. To analyze this association, we obtained long-term follow-up data from a large sample of patients with OHCA; these data were of great value in evaluating the association between psychiatric disorders and long-term mortality.

In the past, there were no specific guidelines for the prevention and treatment of psychological disorders in patients with critical illness or following OHCA. However, the Society of Critical Care Medicine’s International Consensus Conference recently recommended that serial assessments for postintensive care syndrome–related problems continue for 2 to 4 weeks after hospital discharge and should be prioritized among high-risk patients using identified screening tools to prompt referrals for services or more detailed assessments.^32^ Recent studies on the rehabilitation requirement for patients who survive cardiac arrest have been performed, and guidelines for the rehabilitation of patients with critical illness have been published.^14,16,31^ Peskine et al^31^ advised that a specific rehabilitation program for patients following OHCA or patients at risk of impaired functioning is warranted. Based on data from these studies, the recent International Liaison Committee on Resuscitation guidelines emphasize the importance of psychological as well as physical rehabilitation in individuals who survive cardiac arrest.^10^ The ERC and European Society of Intensive Care Medicine guidelines recommends examinations to detect physical and nonphysical impairment in patients after cardiac arrest and, if necessary, prompt rehabilitation.^10^ In addition, a recent American Heart Association guideline introduced a new link in the chain of survival: recovery from cardiac arrhythmias, highlighting for the first time the importance of rehabilitation.^5^ Because the present study identified an association between psychological dysfunction and an increase in long-term mortality, we believe it provides evidence that psychological rehabilitation of patients with OHCA is crucial. In addition, we noted that providing adequate rehabilitation benefited not only the patient's health-related quality of life but also long-term survival.

### Limitations

This study has several limitations. First, the clinical information on patients with OHCA could not be evaluated because this study used claims data from the NHIS. Variables that could affect the outcomes in patients with OHCA, such as shockable rhythm, bystander cardiopulmonary resuscitation, and the duration of cardiac arrest, could not be identified in our study. Second, the results of this study might be biased by potential confounders, such as treatment initiation after depression or anxiety and a lifestyle pattern with low levels of physical activity and appetite and the presence of sleep disturbances. Third, new medical problems and new medications may contribute to the causes of long-term mortality, but we were unable to adjust for all variables. Fourth, because this study was performed using diagnostic codes, it was impossible to include those who did not receive a diagnosis of depression and anxiety disorders because they did not visit the hospital. Fifth, the diagnosis of OHCA and cause of death were defined using *ICD-10* codes, and we cannot rule out diagnostic inaccuracies. In particular, the identification of patients with OHCA and in-hospital cardiac arrest can be inaccurate. We confirmed the definition of OHCA, but the small sample size is a limitation.

## Conclusions

Among patients who survived OHCA, those diagnosed with a psychiatric disorder had a higher long-term mortality rate. The findings of this study suggest that it may be important to provide psychological as well as neurologic rehabilitation to individuals after OHCA to help improve long-term survival.

## Notes

Supplement 1. **eTable 1.** Baseline Characteristics of the Study Population With Depression Disorder **eTable 2.** Baseline Characteristics of the Study Population With Anxiety Disorder **eFigure.** Inverse Kaplan-Meier Curves for Long-term Mortality in OHCA Patients With and Without Depression and Anxiety

Supplement 2.
Data Sharing Statement
