# Supplementary material for: Analysis of Anxiety or Depression and Long-term Mortality Among Survivors of Out-of-Hospital Cardiac Arrest
Source: JAMA Netw Open. 2023 Apr 12;6(4):e237809. doi: 10.1001/jamanetworkopen.2023.7809 (PMC10098954; doi:10.1001/jamanetworkopen.2023.7809)
Supplement: Supplement 1. — eTable 1. Baseline Characteristics of the Study Population With Depression Disorder eTable 2. Baseline Characteristics of the Study Population With Anxiety Disorder eFigure. Inverse Kaplan-Meier Curves for Long-term Mortality in OHCA Patients With and Without Depression and Anxiety [file jamanetwopen-e237809-s001.pdf]

Supplementary Online Content

Lee J, Cho Y, Oh J, et al. Analysis of anxiety or depression and long-term mortality among survivors of out-of-hospital cardiac arrest. *JAMA Netw Open*. 2023;6(4):e237809. doi:10.1001/jamanetworkopen.2023.7809

eTable 1. Baseline Characteristics of the Study Population With Depression Disorder

eTable 2. Baseline Characteristics of the Study Population With Anxiety Disorder

eFigure. Inverse Kaplan-Meier Curves for Long-term Mortality in OHCA Patients With and Without Depression and Anxiety

This supplementary material has been provided by the authors to give readers additional information about their work.

© 2023 Lee J et al. *JAMA Network Open.*

eTable 1. Baseline characteristics of the study population with depression disorder

	Total OHCA survivors (n=2,373)	Patients without depression (n=2,122)	Patients depression (n=251)	p-value
Age category, n (%)				0.230
18-39	426 (18.0)	369 (17.4)	57 (22.7)	
40-49	519 (21.9)	461 (21.7)	58 (23.1)	
50-59	694 (29.2)	625 (29.5)	69 (27.5)	
60-69	432 (18.2)	397 (18.7)	35 (13.9)	
70-79	222 (9.4)	199 (9.4)	23 (9.2)	
≥80	80 (3.4)	71 (3.3)	9 (3.6)	
Sex, n (%)				0.465
Male	1,860 (78.4)	1668 (78.6)	192 (76.5)	
Female	513 (21.6)	454 (21.4)	59 (23.5)	
CCI, n (%)				0.785
0	697 (29.4)	627 (29.5)	70 (27.9)	
1	710 (29.9)	633 (29.8)	77 (30.7)	
2	498 (21.0)	440 (20.7)	58 (23.1)	
3	270 (11.4)	246 (11.6)	24 (9.6)	
≥4	198 (8.3)	176 (8.3)	22 (8.8)	
Long term death	675 (28.4)	581 (27.4)	94 (37.5)	0.001

Categorical variables are represented by number (%). The two groups were compared using chi-square test for categorical variables. CCI, Charlson comorbidity index

© 2023 Lee J et al. *JAMA Network Open.*

eTable 2. Baseline characteristics of the study population with anxiety disorder

	Total OHCA survivors (n=2,373)	Patients without anxiety (n=2,146)	Patients with anxiety (n=227)	p-value
Age category, n (%)				0.504
18-39	426 (18.0)	382 (17.8)	44 (19.4)	
40-49	519 (21.9)	471 (21.9)	48 (21.1)	
50-59	694 (29.2)	622 (29.0)	72 (31.7)	
60-69	432 (18.2)	394 (18.4)	38 (16.7)	
70-79	222 (9.4)	200 (9.3)	22 (9.7)	
≥80	80 (3.4)	77 (3.6)	3 (1.3)	
Sex, n (%)				0.310
Male	1,860 (78.4)	1688 (78.7)	172 (75.8)	
Female	513 (21.6)	458 (21.3)	55 (24.2)	
CCI, n (%)				0.756
0	697 (29.4)	637 (29.7)	60 (26.4)	
1	710 (29.9)	641 (29.9)	69 (30.4)	
2	498 (21.0)	451 (21.0)	47 (20.7)	
3	270 (11.4)	240 (11.2)	30 (13.2)	
≥4	198 (8.3)	177 (8.2)	21 (9.3)	
Long term death	675 (28.4)	603 (28.1)	72 (31.7)	0.247

Categorical variables are represented by number (%). The two groups were compared using chi-square test for categorical variables. CCI, Charlson comorbidity index

© 2023 Lee J et al. *JAMA Network Open.*

eFigure. Inverse Kaplan-Meier Curves for Long-term Mortality in OHCA Patients With and Without Depression and Anxiety

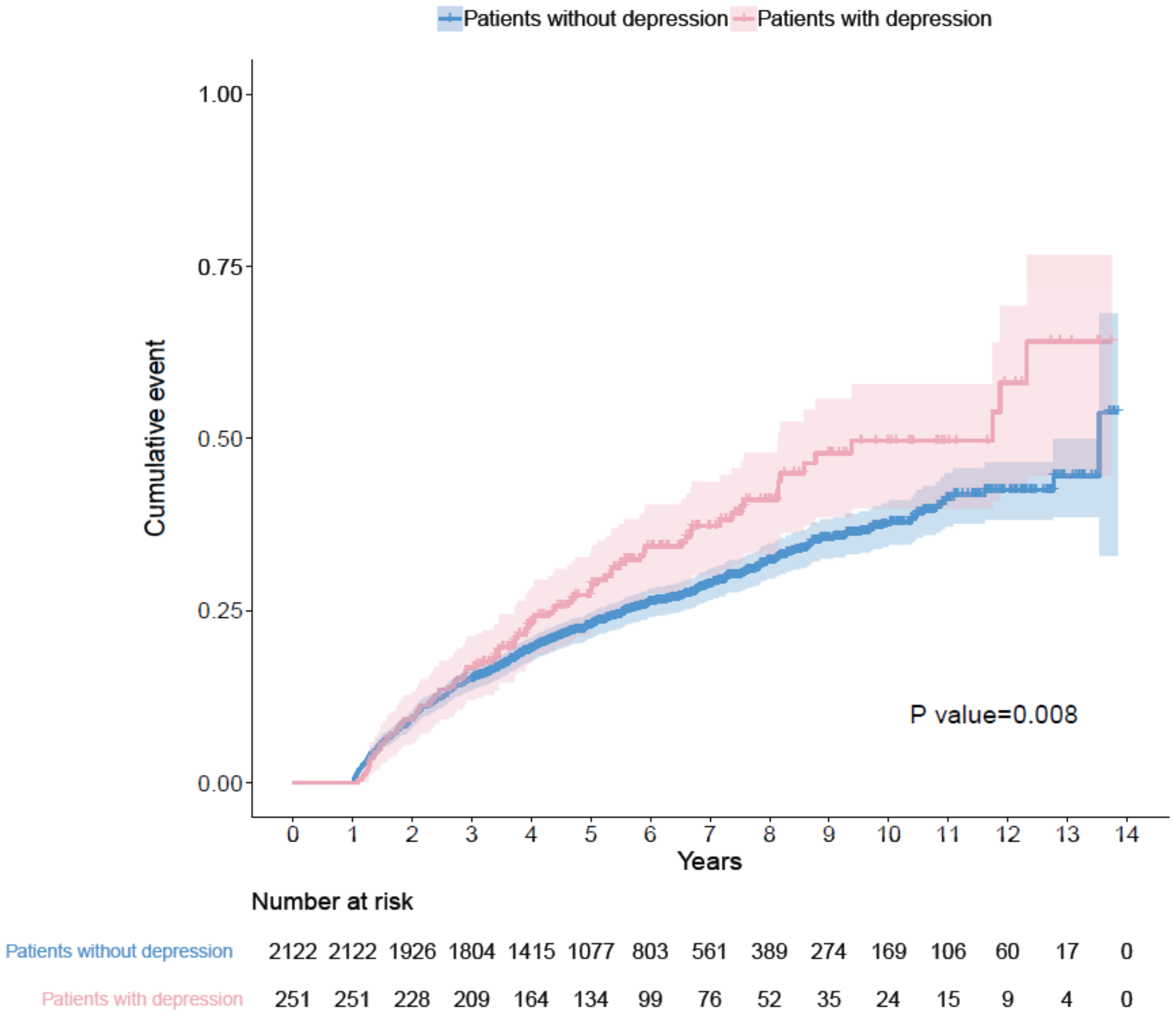

A. Inverse Kaplan–Meier curves for long-term mortality in OHCA patients with and without depression. Patients without depression (n=2,122), Patients with depression (n=251)

Since patients who survived more than 1 year after OHCA were targeted, the first year was excluded from the graph.

© 2023 Lee J et al. *JAMA Network Open.*

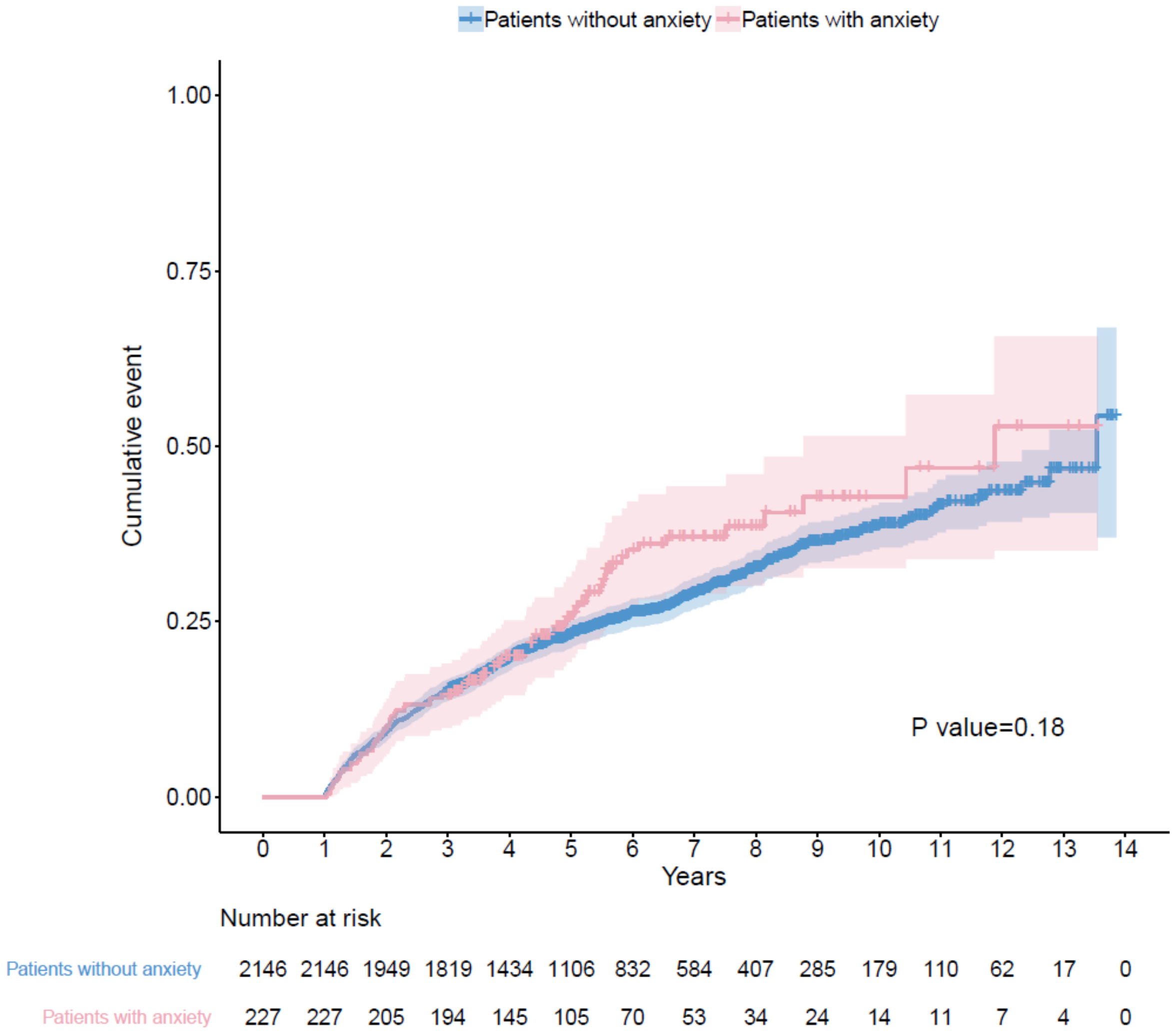

B. Inverse Kaplan–Meier curves for long-term mortality in OHCA patients with and without anxiety. Patients without anxiety (n=2,146), Patients with anxiety (n=227)

Since patients who survived more than 1 year after OHCA were targeted, the first year was excluded from the graph.

© 2023 Lee J et al. *JAMA Network Open.*
