# Supplementary material for: Analysis of Anxiety or Depression and Long-term Mortality Among Survivors of Out-of-Hospital Cardiac Arrest
Source: JAMA Netw Open. 2023 Apr 12;6(4):e237809. doi: 10.1001/jamanetworkopen.2023.7809 (PMC10098954; doi:10.1001/jamanetworkopen.2023.7809)
Supplement: Supplement 2. — Data Sharing Statement [file jamanetwopen-e237809-s002.pdf]

Data Sharing Statement

Lee. Analysis of Anxiety or Depression and Long-term Mortality Among Survivors of Out-of-Hospital Cardiac Arrest. *JAMA Netw Open*. Published April 12, 2023. doi:10.1001/jamanetworkopen.2023.7809

Data

Data available: National Health Insurance Service (NHIS) data are third-party data that are not owned by the authors. The raw data can be accessed with permission from the NHIS in Korea.
